# Plasmonic hot spots reveal local conformational transitions induced by DNA double-strand breaks

**DOI:** 10.1038/s41598-022-15313-4

**Published:** 2022-07-15

**Authors:** Sara Seweryn, Katarzyna Skirlińska-Nosek, Natalia Wilkosz, Kamila Sofińska, David Perez-Guaita, Magdalena Oćwieja, Jakub Barbasz, Marek Szymoński, Ewelina Lipiec

**Affiliations:** 1 M. Smoluchowski Institute of Physics, Jagiellonian University, Łojasiewicza 11, 30-348 Kraków, Poland; 2 Department of Analytical Chemistry, University of Valencia, 50 Dr Moliner Street, 46100 Burjassot, Spain; 3 Jerzy Haber Institute of Catalysis and Surface Chemistry, Polish Academy of Sciences, Niezapominajek 8, 30‑239 Kraków, Poland

**Keywords:** Biochemistry, Biophysics, Molecular biology

## Abstract

DNA double-strand breaks (DSBs) are typical DNA lesions that can lead to cell death, translocations, and cancer-driving mutations. The repair process of DSBs is crucial to the maintenance of genomic integrity in all forms of life. However, the limitations of sensitivity and special resolution of analytical techniques make it difficult to investigate the local effects of chemotherapeutic drugs on DNA molecular structure. In this work, we exposed DNA to the anticancer antibiotic bleomycin (BLM), a damaging factor known to induce DSBs. We applied a multimodal approach combining (i) atomic force microscopy (AFM) for direct visualization of DSBs, (ii) surface-enhanced Raman spectroscopy (SERS) to monitor local conformational transitions induced by DSBs, and (iii) multivariate statistical analysis to correlate the AFM and SERS results. On the basis of SERS results, we identified that bands at 1050 cm^−1^ and 730 cm^−1^ associated with backbone and nucleobase vibrations shifted and changed their intensities, indicating conformational modifications and strand ruptures. Based on averaged SERS spectra, the PLS regressions for the number of DSBs caused by corresponding molar concentrations of bleomycin were calculated. The strong correlation (R^2^ = 0.92 for LV = 2) between the predicted and observed number of DSBs indicates, that the model can not only predict the number of DSBs from the spectra but also detect the spectroscopic markers of DNA damage and the associated conformational changes.

## Introduction

Every cell of the human body is continuously exposed to damaging factors, such as ionizing radiation, free radicals, and other mutagenic agents. This constant assault on DNA generates tens of thousands of DNA lesions per day^1^. DNA damage may also occur because of spontaneous chemical reactions or interactions with a plethora of chemical substances, including anticancer drugs such as bleomycin (BLM). Chemicals may cause modifications of base pairs, pyrimidine dimers, and the formation of single- or double-strand breaks (SSBs and DSBs, respectively). DSBs are the most dangerous type of DNA damage, often resulting in genetic instability. Untreated or incorrectly repaired DSBs can block genome replication and transcription, leading to cellular death, translocations, or other mutations that induce carcinogenesis and other pathologies^2^. To maintain genomic integrity in all life forms, this kind of DNA damage must be repaired.

In this work, we present the research on structural modifications in the DNA molecule induced by an anticancer antibiotic^3^ bleomycin. This drug is known to cause both SSBs and/or DSBs. DNA cleavage results from the binding of the bleomycin-iron complex to the DNA helix in the presence of oxygen^4^. It was determined that the most probable sites of cuts inducted by bleomycin are 5′-G-pyrimidine (5′-GT and 5′-GC)^5^. However, for purified DNA, also the dinucleotides GG, GA, AT, AC, and AA were observed to be cleaved by bleomycin^6^. Further studies performed by Murray et al.^6–8^ revealed that the flanking sequence of cleaved dinucleotides may influence the intensity of the BLM cleavage of DNA. The next-generation DNA sequencing enabled to establish of the extended DNA sequence, which is the most frequently cleaved by bleomycin resulting in DSBs formation. The extended genome-wide DNA sequence specificity of bleomycin cleavage was established to be 5′-TGTAW (where W is T or A) for purified human DNA, and 5′-GTGTA for cellular DNA^6,8,9^. The differences in obtained results for purified and cellular DNA were attributed to the cellular environment, and a presence of e.g., proteins that may interact with DNA altering its conformation, and thus, the ability to interact with bleomycin. Moreover, the supercoiled nature of cellular DNA may influence the ability to interact with BLM at particular sequences^6^. However, for both purified and cellular DNA, the core sequence cleaved by bleomycin was 5′-TGTA^6^. The exact mechanism of DSBs induction by BLM is still not fully understood^10^. It seems that not only the mechanism of action of the BLM but also its influence on the local DNA conformational transitions is not clear.

DNA conformational transitions were found to be implicated in DNA damage and repair processes^11^. The majority of DNA in functional cells is mainly in the B-DNA form^12^. DNA may also fold into A-DNA and Z-DNA conformations^13^. All these DNA forms differ in the dimensions of the major and minor grooves^12^. A-DNA has more base pairs (bps) per turn of the helix compared to B-DNA. Thus, A-DNA is more compressed along its axis, and its length is 20–25% shorter than the B-DNA considering the same number of bps. Alternating purine/pyrimidine sequences (CGCGCG) favour the left-handed Z-DNA form^12^. Specific DNA sequences are known to favour its particular conformations, for example, AGCTTGCCTTGAG forms A-DNA, CGCGAATTCGCG is specific for B-DNA helix, and CGCGCGTTTTCGCG favours Z-DNA conformation^14,15^. The structural properties determine the ability of DNA to react with other macromolecules. The locally induced A-DNA form has already been identified in some protein-DNA complexes^16^. Moreover, it is presumed that local B-to-A-DNA conformational transition occurs in the damaged area of DNA^12^. Conformational rearrangements of DNA molecules were studied by Seo et al.^17^ using fluorescence-based approaches to detect directly DSBs in human cells. Recently, super-resolving fluorescence microscopy, specifically, fluctuation-assisted binding-activated localization microscopy (fBALM), was used for the first time to assess differences in the nuclear architecture at the nanoscale^18^. Many techniques are available for detecting and characterizing DNA, including molecular spectroscopy, such as Raman or infrared spectroscopy^19^; however, mainly because of the insufficient sensitivity, they cannot detect the local structural modifications of DNA molecules after the induction of DSBs^20^. Therefore, the application of one of the most sensitive analytical techniques, such as surface-enhanced Raman spectroscopy (SERS), may help to overcome this limitation of conventional spectroscopies.

SERS enhances Raman scattering using metallic nanostructures at the supporting surface, such as metallic nanoparticles^21^. The enhancement of the cross-section for Raman scattering mainly results from the additional electromagnetic field that is induced by the collective movements of electrons in nanoparticles under the influence of the external electromagnetic field of the Raman laser light. This additional electromagnetic field increases the sensitivity of the method and allows the observation of molecular changes that cannot be detected by conventional Raman spectroscopy^22^. The effect of electromagnetic enhancement depends mainly on the type and properties of plasmonic nanostructures. A major limitation of SERS is related to low spectral reproducibility induced by inconsistent enhancement due to a challenging synthesis of reproducible nanoparticles and their controlled aggregation^23^. The development and use of highly reproducible nanostructures are crucial in the application of SERS as a mainstream spectroscopic technique^24^.

Over the past decade, SERS became a powerful analytical technique in chemistry, material science, and biotechnology. It provides ultra-high sensitivity and intrinsic chemical fingerprint information; thus, conformational and structural changes in DNA at very low concentrations of analyte can be investigated^25^. To date, various studies of DNA damage have been performed. Panikkanvalappil et al*.*^26^ detected via SERS DSBs induced by reactive oxygen species (ROS) after the exposure of DNA to H_2_O_2_/UV. The authors assumed that a split of the peak at 740 cm^−1^ into two peaks at 715 cm^−1^ and 738 cm^−1^ indicates unpaired adenine and thymine residues resulted from DNA strand breaks. A study of the ROS effect on the DNA damage induction upon photodynamic therapy (PDT) treatment was presented by Yue et al.^27^. An intensity decrease and a blueshift of the phosphate symmetric stretching band at 1092 cm^−1^ were correlated with conformational changes of DNA^27^. Guselnikova et al.^28^ investigated the UV-induced DNA damage. The Raman marker bands related to the chemical modifications of DNA, typical for UV-induced DSBs, were assigned. Nitrogenous ring stretching and vibrations of amino groups were attributed to chemical changes in heterocyclic DNA fragments, contributing to the formation of cyclobutane pyrimidine dimer (CPD) and other photoproducts. The symmetric stretching of the phosphate groups was associated with environmental transformation, which proved the CPD formation or oxidation of the sugar part. Ou et al.^29^ applied SERS to study the influence of X-ray radiation on genomic DNA treated with various radiation doses (6, 10, 15, and 20 Gy) and under different cell incubation times (0, 24, 48, and 72 h)^29^. Chemical modifications of DNA induced by chemotherapeutic drugs, e.g., doxorubicin, were investigated with different SERS enhancement factors and various SERS configurations^30^. Barhoumi et al.^31^ applied SERS to monitor the influence of cisplatin on DNA structure^31^. Cisplatin is known to create covalent bonds with DNA, leading to DNA replication disorders and cell apoptosis. The effect of the cisplatin on DNA was also studied by Masetti and co-workers^32^. The authors revealed that the appearance of the peak at 541 cm^−1^ indicates the formation of a covalent bond between Pt (cisplatin) and N atoms from guanine^32^.

On the other hand, a significant aspect of research on DNA damage is a precise estimation of the number of lesions. Gel electrophoresis is commonly applied in the qualitative detection of DSBs. However, quantitative estimation of the number of strand breaks based on the results of gel electrophoresis is prevented or largely prohibited due to methodological limitations. According to the literature, gel electrophoresis lacks in the resolution to quantify relatively short DNA fragments, sampling only relatively large DNA fragments^33,34^.

**Figure 1:**
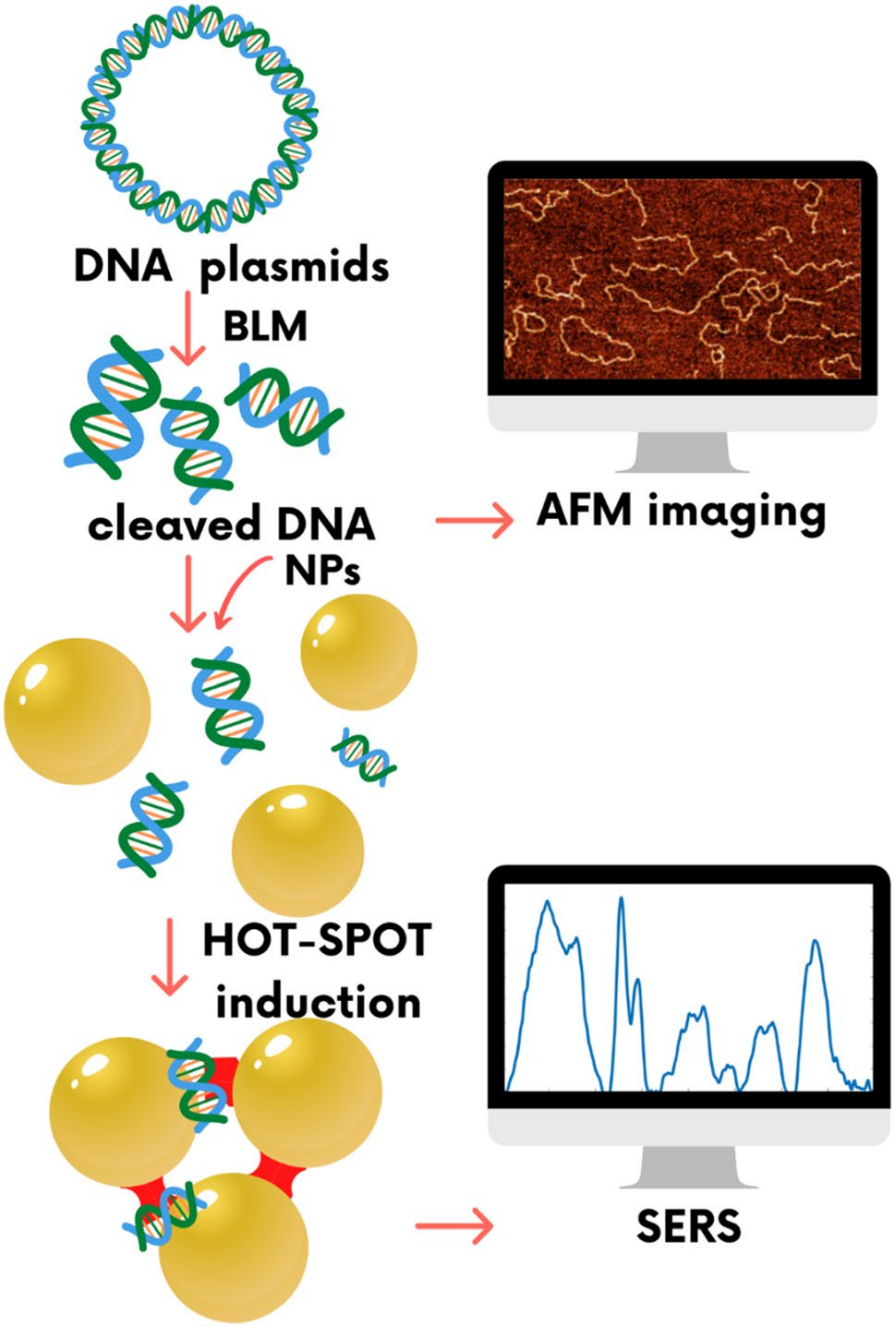
Scheme of concept of SERS and AFM experiments. The applied approach combines two independent experimental methods, SERS and AFM, in the detection of the DNA damage resulting from the induction of DSBs by bleomycin. AFM enables a visualization of the effect of DNA cutting by bleomycin, while SERS is sensitive enough to detect conformational rearrangements of DNA resulting from DSBs induction via bleomycin.

In this work, we combined atomic force microscopy (AFM) to visualize the products of DNA cleavage induced by BLM with their chemical characterization by SERS. Although AFM itself clearly demonstrates the effect of BLM action on DNA molecules, visualized as the decreasing length of DNA fragments with increasing BLM concentration, it does not provide any information regarding the chemical structure of the damaged DNA. Thus, we incorporate a SERS-based method for monitoring of local conformational changes in BLM-treated DNA. As an enhancement factor, we used gold nanoparticles stabilized with cysteamine. Moreover, we apply a statistical model allowing simultaneous analysis of AFM and SERS results. A schematic representation of our experimental concept is presented in Fig. 1.

## Results and discussion

### AFM imaging and DNA double-strand breaks induced via bleomycin

**Figure 2:**
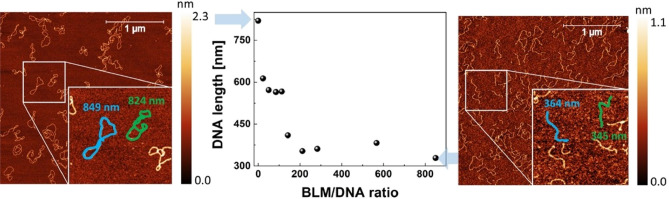
Average length of DNA fragments [nm] versus BLM/DNA molar ratio (statistical details and typical AFM images of DNA treated with various concentrations of BLM are presented in Supplementary Table S1 and Supplementary Fig. S1 respectively). AFM images of the pUC19 DNA plasmid deposited on mica indicating untreated (control) DNA (left) and DNA treated with an 850 BLM/DNA molar ratio (right). In the magnified area of AFM topographies, the concept of DNA molecule length tracing is schematically shown.

The average length of DNA fragments versus the increasing molar ratio of BLM molecules per DNA plasmid is plotted in Fig. 2. According to our results, the average length of the control (undamaged) DNA plasmid is 820 nm, which is comparable to the expected length of the DNA strand containing 2686 bp (pUC19 plasmid), which is ~ 870 nm, assuming 3.4 nm per 10.5 bp for B-type DNA^35^. With the increase in bleomycin content in the samples, the average fragment lengths become shorter. In the range of 0–200 BLM/DNA molar ratios, we detected a sharp decrease in the average length followed by a flattened depression for higher concentrations of BLM (above a BLM/DNA molar ratio of 200). The decrease in average DNA length indicates an increasing number of DSBs. The observed flattening of the dependence may be related to local conformation changes at cleavage sites prohibiting further interaction of bleomycin with DNA. This is due to the limited access caused by changes in the size of DNA grooves. AFM images of DNA treated with various concentrations of BLM are presented in the Supplementary Information (Fig. S1).

### Molecular spectroscopy (SERS and RAMAN) of DNA treated with bleomycin

**Figure 3:**
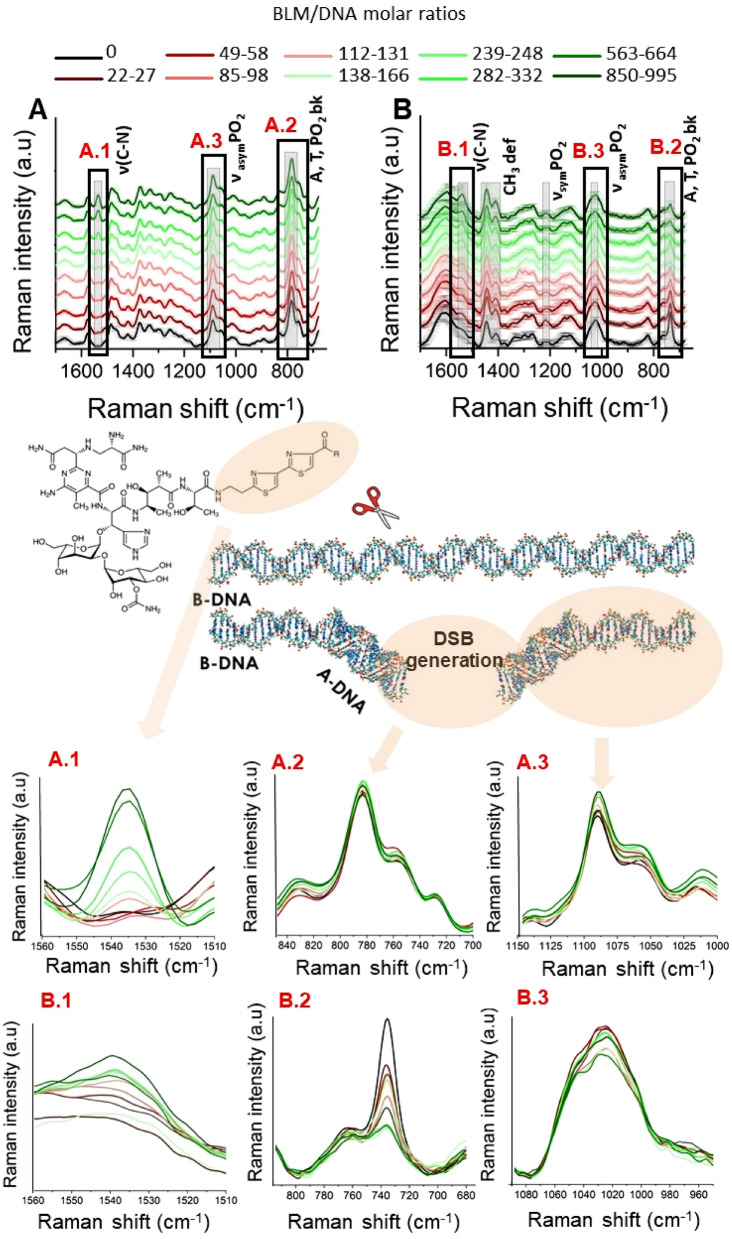
Molecular spectroscopy of DNA treated with bleomycin (**A**) Raman and (**B**) SERS spectra of DNA solutions: control and BLM treated, with the magnified area of the Raman band at 1535 cm^−1^ corresponding to bithiazole moiety present in the BLM molecule (**A.1**,**B.1**), C5′–O–P–O–C3′ phosphodiester bonds band at 850–680 cm^−1^ (**A.2**,**B.2**) and symmetric stretching of phosphate at 1100–960 cm^−1^ (**A.3**,**B.3**) and the spectral ranges of nucleobases (A, T). The three bands are related to the presence of BLM (**A.1**,**B.1**), breaks of DNA backbone (**A.2**,**B.2**) and partial conformational transition of DNA (**A.3**,**B.3**) respectively.

Raman spectra of the control DNA sample and DNA treated with bleomycin are shown in Fig. 3A. Because of the lack of sensitivity, the only observable difference between the spectra of the control and damaged DNA is the band at 1535 cm^−1^ assigned to the bithiazole moiety^36^, which appears in the spectra acquired from DNA treated with BLM at a BLM/DNA molar ratio of 112 and higher concentrations of BLM (as visible in the magnified spectral range in Fig. 3A.1). However, no statistically significant differences were observed in the spectral ranges related to DNA backbone bands, which are a hallmark of expected conformational change (Fig. 3A.2,A.3). In contrast, the SERS spectra show differences in the bithiazole moiety from (Fig. 3B1) and evidence of variability in the DNA regions (Fig. 3B2,B3). These results clearly show that although both techniques are closely related, they provide different information. While Raman is affected by the concentration of BLM, SERS bands indicate changes in the DNA conformation. Raman shifts and differences in intensity of the vibrational modes in SERS spectra in comparison to the standard Raman spectra are due to the use of AuNPs. When the molecule is close to the nanostructured substrate, its symmetry might change slightly, as well as the selection rules. Therefore, the different sections of the spectrum can be enhanced differently^37^. The observed spectral positions of the Raman and SERS bands for DNA molecules and BLM are shown in Fig. S2 of the Supplementary Data.

The high enhancement of the Raman signal achievable in SERS^38^ enables monitoring of the DNA chemical modifications induced by strand breaks. SERS spectra are generated locally from SERS hot spots located between nanoparticles. According to the dependence of the diffusion transport rate on the mass of objects, the shortest DNA fragments diffuse to the surface of nanoparticles first^39^. Since the expected conformational change is local and involves only the DNA backbone at the lesion site, the relative abundance of the changed DNA conformation is higher in short DNA fragments than in long strands. Therefore, in contrast to Raman spectroscopy, SERS is especially sensitive for probing local conformational changes in DNA. In Raman spectroscopy, the acquired signal is averaged from the whole bulk of DNA molecules. The signal from the unmodified backbone is dominant, and local conformational changes cannot be detected. SERS spectra acquired from damaged DNA were compared with spectra of the control DNA sample and then with Raman experiments. The major limitation of SERS is related to the unstable enhancement of the electromagnetic field, which affect the reproducibility of the acquired spectra. We overcome this difficulty by acquisition of a relatively large number of spectra (several hundreds). Moreover, nanoparticles or the aggregating agent (NaCl in our article) may interact with the analyte and affect its molecular structure. We took this factor into consideration and tested various concentrations of salt and nanoparticles, as well as applied stabilizers as presented in Supplementary Information (Figs. S4–S6).

Averaged SERS spectra of the DNA-BLM complex are shown in Fig. 3B. Standard deviations are also demonstrated. The obtained SERS spectra exhibit high reproducibility and intense marker bands typical of DNA. Some of them include the vibration of nucleobases, and C5′–O–P–O–C3′ phosphodiester bonds appear at 734 cm^−1^^40^. The Raman bands at approximately 1024 cm^−1^ and 1215 cm^−1^ are assigned to the symmetric and asymmetric stretching of phosphate groups from the DNA backbone, respectively^41^, and deformation vibrations of methylene groups appear at 1407 cm^−1^ and 1444 cm^−1^^42^. Detailed band assignments are presented in Supplementary Table S2. In general, Raman bands between 1100 and 800 cm^−1^ are correlated with the backbone geometry and are sensitive to secondary structure modifications of DNA^43^. Differences between SERS spectra acquired from the DNA treated with BLM and control—untreated DNA were observed mainly in the phosphate backbone marker bands. We noted that as the concentration of bleomycin in DNA solution increased, the peak at 1024 cm^−1^ was split into two peaks and slightly blue-shifted (Fig. 3B3). The observed changes in the spectral shape of this band can be attributed to partial conformational modifications of DNA, specifically, the partial conformational transition from B-like-DNA to other DNA forms, such as A-DNA or Z-DNA^12,44^, followed by the appearance of double- or single-strand breaks resulting from the DNA-bleomycin interaction. This attribution was supported by a detailed analysis of data recorded from the spectral range referring to characteristic Raman bands of C5′–O–P–O–C3′ phosphodiester bonds and nucleobases (Fig. 3B.2). In fact, the SERS spectrum obtained for the control DNA solution exhibited an intense Raman band at 734 cm^−1^, which corresponds to adenine ring breathing, and thymidine vibrations merged together^26^. As a consequence of BLM-induced DNA strand cleavage, the nucleobases become unstuck, and thus, the intensity of the band observed at 734 cm^−1^ decreases. With an increase in the molar content of the damaging factor, a new vibration band at 749 cm^−1^ is observed and can be assigned to unpaired thymidine residues^26^. Moreover, the Raman band at 734 cm^−1^ is also assigned to phosphodiester bond vibrations. Here, the decrease in intensity is associated with strand breaks resulting from interruptions within the C5′–O–P–O–C3′ bond^45^. For more detailed information, second derivatives were computed and attached in Fig. S3 in the Supplementary Data.

**Figure 4:**
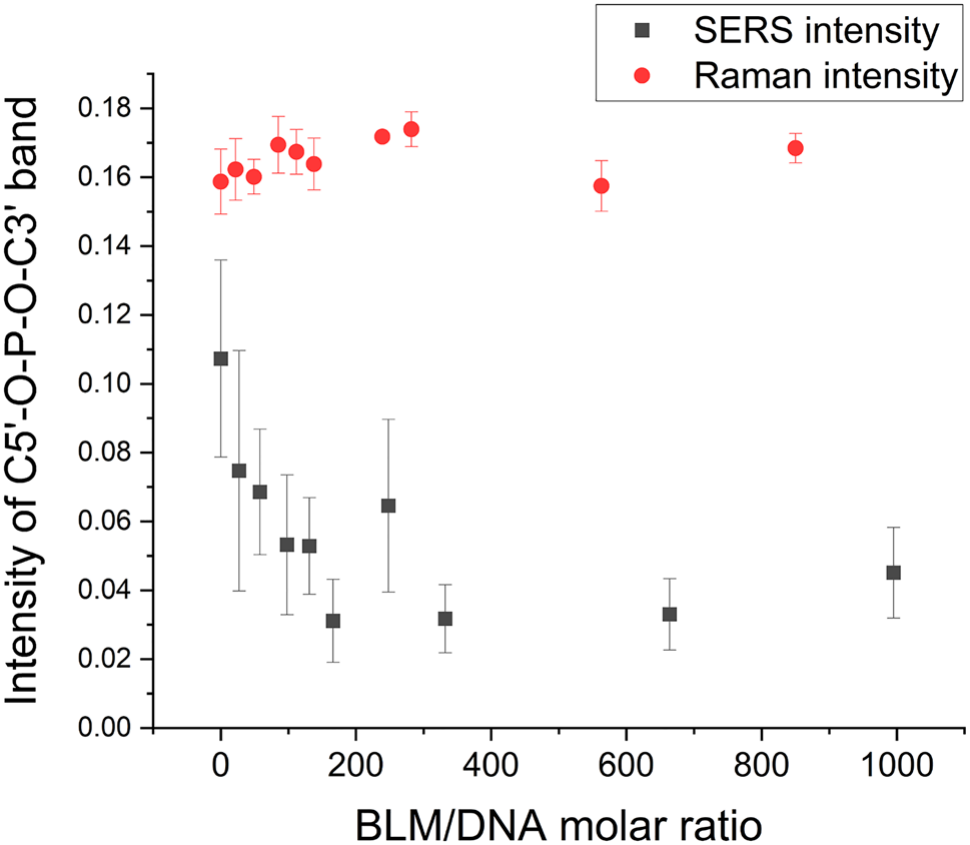
Comparison of the intensity of the C5′–O–P–O–C3′ band versus the BLM/DNA molar ratio probed by SERS and Raman spectroscopy.

To demonstrate the high sensitivity of SERS for the detection of molecular changes induced by DNA damage, we compared the SERS and Raman intensities of the C5′–O–P–O–C3′ stretching band at 734 cm^−1^ for SERS and 783 cm^−1^ for Raman (Fig. 4). The intensity of the C5′–O–P–O–C3′ band attributed to phosphodiester bonds and nucleobases decreased with the BLM concentration in the SERS spectra, whereas in the Raman spectra, no changes were observed. For SERS, an intensity decrease is observed over BLM/DNA molar ratios of 0–200. For higher concentrations of BLM, the intensity of this band remains constant. This observation is consistent with the results of AFM imaging, which demonstrated the constant average length of DNA fragments above a BLM/DNA molar ratio of ca. 200. This result suggests that an increase in the BLM/DNA molar ratio above 200 does not induce more DNA cuts. However, each DSB may induce local conformation changes at the lesion site^46^. As a consequence, after certain finite DSB events, DNA fragments containing potential targets for bleomycin, including purine/pyrimidine sequences including 5′-GT and 5′-GC^5^, may adopt conformations A or Z, which prohibits or largely prevents further interaction between BLM and DNA. BLM interacts with the B-DNA form only via the minor groove^47^. Therefore, bleomycin molecules no longer have spatial access to their potential targets due to the geometrical limits related to the changed size of minor grooves in the A or Z conformations in comparison to the B-DNA form^16^. Hence, despite the molar excess of bleomycin, DSBs can no longer be created.

Our previous studies proved that the nature of a single DSB cannot influence the SERS and Raman spectra itself due to an insufficient sensitivity prohibiting probing of individual broken bands^48^. Tip-enhanced Raman scattering allows characterization of broken bands in contrast to Raman or SERS^48^. However, as we presented also in this article, SERS enables monitoring of the effect related to the induction of DSBs on the structural rearrangement of DNA (e.g., its conformation). In addition, an advantage of SERS in comparison to TERS is that the signal is acquired from a bulk sample solution and the direct influence of the substrate surface on the molecular structure of the analyte is avoided. To demonstrate the broader utility of the presented methodology we have applied it to follow conformational change caused by physical damaging factors specifically photons. UVC light applied as a damaging factor inducing DSBs enabled to avoid additional bands in the SERS spectrum related to the presence of chemicals^48^. Additionally, we have demonstrated the capability of our approach in studies of BLM influence on ca 30 kbp fragments of genomic DNA isolated from Jurkat cells. The observed spectral changes related to DSBs formation, and the DNA conformational changes were consistent with those described here. The results of these additional experiments are presented in the Supplementary Information (Figs. S10 and S11).

### Statistical analysis

**Figure 5:**
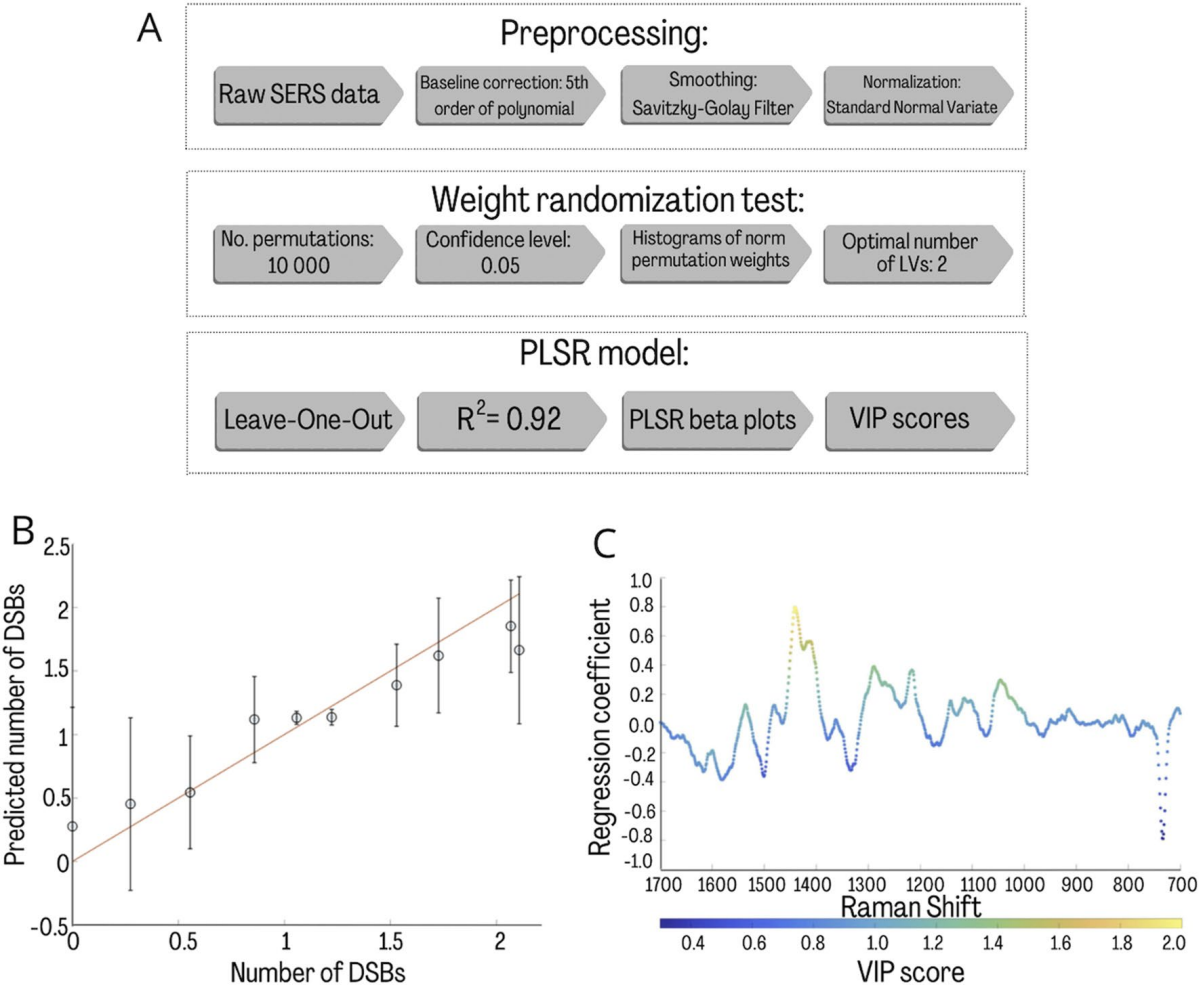
(**A**) A schematic representation of the analysis performed on the raw SERS spectra to build the PLSR model. The obtained values for each analysis step are presented. PLSR model for the number of DSBs. (**B**,**C**) Predicted versus observed values calculated based on AFM images according to the formula described in the “Materials and methods” section^50^ (**B**) and regression coefficient (β) plot (**C**). Averaged spectra of the corresponding parameter were used as the PLSR model input.

PLSR model for prediction of the DSBs number. The PLSR aimed to model the number of DSBs (Y) induced by bleomycin molar concentration from the averaged Raman spectra (X). To determine the optimal number of latent variables (LVs), a weight randomization test (10,000 permutations, confidence level = 0.05) was performed^49^. Leave-one-out cross-validation was employed to test the generalization power of the model. Finally, the regression coefficient (PLSR β) and the VIP scores were used to investigate spectral features associated with the regression, providing spectral information about the biochemical changes related to the number of DSBs. Figure 5 presents the overall concept of data processing performed during this analysis.

Figure 5 shows the results from the PLSR model. The strong correlation (R^2^ = 0.92 for LV = 2) between the predicted and observed number of DSBs is prominent in Fig. 5B, indicating that the model could predict the number of DSBs from the spectra. This finding clearly indicated that the SERS spectra hold significant information about the number of DSBs in the sample. The regression coefficients (PLSR β) calculated for this model are presented in Fig. 5C. All distinct features present in the PLSR β plots were assigned according to the literature and gathered in Table S3 in Supplementary Information. The VIP values were generated from the model and are presented in Fig. 5C as a color scale. To determine the level of contribution of specific frequencies in discrimination, the bands were compared with the VIP values. This approach is commonly used because of the relevance of quantitative indication of specific band assignments in analysis.

Figure 5C shows the changes in DNA conformation caused by the interaction with bleomycin that can be predicted by the described model. For instance, Raman bands related to ring breathing vibrations at 785 cm^−1^ and symmetric PO_2_ stretches at 1080 cm^−1^ are observed. The significant VIP value at 1264 cm^−1^ is related to the asymmetric PO_2_ stretch and is also due to changes in the DNA backbone conformation. The important contribution to the model is also related to the DNA conformation marker presented at 1438 cm^−1^ and 1129 cm^−1^ and the triplets of 970 cm^−1^, 951 cm^−1^, and 921 cm^−1^ that determine the Z-form of the DNA. The enhanced peak at 1069 cm^−1^ also suggests this fact. A significant VIP value was also noticed for the ring vibrations of adenine and cytosine, which were affected by bleomycin. The PLSR β plots also show spectral features associated with the ring breathing vibrations of cytosine (789–785 cm^−1^) and adenine (728 cm^−1^), which may suggest the induction of ring modifications by bleomycin.

## Conclusions

Our results proved the high capability of the applied unique multimodal approach, which combines two independent experimental methods, SERS and AFM, with PLSR analysis in the detection of DNA damage and associated molecular modifications—conformational rearrangements. Moreover, the application of the statistical analysis allowed us to explore spectroscopic markers of DNA damage and the associated conformational changes and to detect these structural transitions for the exact number of DSBs calculated based on AFM imaging results.

PLS regressions for the number of DSBs caused by various molar concentrations of bleomycin were calculated based on averaged SERS spectra. The created model presents a strong correlation between the predicted and observed responses. To determine the level of contribution of bands in the analysis, the PLSR β and VIP values were calculated. Raman bands attributed to the ring breathing vibrations of adenine and cytosine may suggest that bleomycin induces not only strand breaks but also ring modifications. Furthermore, the symmetric PO_2_ stretches also have a significant impact on the analysis, suggesting changes of DNA conformation and oxidation of nucleic acids. This research shows that SERS studies of DNA treated with bleomycin are sensitive enough to discriminate the local changes in the DNA conformation that cannot be detected with conventional spectroscopic tools because of the insufficient sensitivity.

The results of two independent experimental techniques demonstrated that DNA cleavage and associated conformational changes were observed for a certain range of BMLM/DNA molar ratios (up to 200), and a further increase in bleomycin concentration did not induce more DNA cleavage. SERS results indicate that this may be related to the local conformational change, which prevents further interaction between bleomycin and DNA.

Here we applied the simplified model of plasmid DNA treated with BLM. An application of AFM to evaluate the number of DSBs in genomic DNA extracted from mammalian cells is challenging because of three reasons: (i) high risk of DNA damage induction and its molecular modifications upon extraction and purification procedures, (ii) difficulties in visualization DSBs in bundles of overlapping DNA strands, that are normally observed on AFM images of DNA isolated from cells^51^, and (iii) methodological limitation of AFM, which indeed provides high-resolution images but does not allow complete visualization of long DNA strands (isolated from cells). Therefore, these technological challenges limited the scope of the present study to plasmid DNA or well purified fragments of genomic DNA with a defined number of base pairs. It constitutes a proof-of-concept study for future experiments. To transfer the scientific problem discussed here to more complex systems like mammalian cells, a different experimental methodology needs to be applied.

In this article, we have investigated the interaction between DNA strands and bleomycin, which results in the induction of DSBs, and discussed the importance of the local conformational transition for DNA repair process. However, the applied and significantly simplified, model system (DNA + BLM) does not allow to study of the kinetics of DNA repair. This process can be followed in living cells after damaging factor treatment. Molecular spectroscopy such as Raman and infrared can be applied to monitor molecular modifications related to DNA repair due to its chemical selectivity and non-invasive nature. Specific fluorescent markers are commonly used to visualize the presence of H2A.X histone phosphorylation, which is a hallmark of DSBs repair^52^. An important aspect that must be taken into consideration in studies of the kinetics of DNA repair, is the differentiation between molecular changes induced only by DNA damage and the ones related to various pathways of DNA repair. To study these processes an inhibition of selected DSB repair pathways is required^53^.

The approach applied in this article combining SERS, AFM, and multivariate statistical analysis was proved to be an efficient tool in correlating the conformational transition B- to A-DNA with the decreasing average length of DNA fragments under the bleomycin treatment. Such an approach could be applied in future research on the influence of various drugs with poorly known mechanisms of action on the DNA structure and conformation. However, while the applied methodology directly links the rate of DSBs with the spectral pattern attributed to A-DNA conformation, it does not provide information regarding the rate of B to A conformational rearrangement, or how far does it reach from the DSBs site. To address this issue, highly sensitive techniques that provide local chemical information are required. Future studies on the conformational rearrangements of the DNA under the influence of external factors (e.g., drugs) will involve molecular nanospectroscopies, specifically tip-enhanced Raman spectroscopy (TERS) or infrared nanospectroscopies to probe the DNA structure with the spatial resolution of single nanometers.

## Materials and methods

The preparation procedure and the physicochemical characteristic of AuNPs are described in the Supplementary Information, supporting data is presented in Figs. S7–S9.

### Bleomycin preparation

Bleomycin sulphate was obtained from TCI Europe N.V. (Tokyo Chemical Industry). Activated bleomycin for all experiments was prepared by first dissolving ferric ammonium sulphate dodecahydrate in water and then immediately adding a 10% molar excess of iron to bleomycin^54^. 1 mM bleomycin-iron solution was kept at − 20 °C until use.

### SERS measurements

For SERS investigation of BLM-induced molecular modifications in the DNA, 1.3 µL of bleomycin was added to a 1.2 µL DNA solution (500 mg L^−1^). The pUC19 DNA plasmid for all experiments was purchased from Thermo Fisher Scientific Inc. (NYSE: TMO). The concentration of the bleomycin solution was 7, 15, 25.5, 34, 43.2, 64.6, 86.5, 173, and 259.4 µM to achieve a series of solutions with BLM/DNA molar ratios from ca. 20–1000. After 4 min, a 2.5 µL AuNP suspension (176 mg L^−1^) was added. To obtain the ionic strength of 3.6 × 10^–2^ M in the prepared mixture, after 1 h incubation, 0.7 µL NaCl (0.3 M) was added. The abundance of the salt ions in the solution defines the Debye length. Therefore, by increasing the ionic strength of the solution, the distance between particles can be reduced, creating areas of strong enhancement of the electromagnetic field, so-called hot spots, in gaps between the particles. The salt concentration was chosen to obtain a Debye length of *κ*^−1^ = 1.6 nm as the value for the greatest enhancement for probing the molecular structure of DNA. After 30 min, a 1 µL droplet of this prepared sample was deposited on clean aluminium foil for immediate SERS spectra acquisition before sample drying to avoid dehydration-related conformational transitions. A confocal Raman system (LabRAM HR, Horiba France SAS) with a BX41 upright optical microscope (Olympus) was applied for data acquisition. A 633 nm laser light was used for excitation, and the laser beam was focused onto the sample by a 50 × objective. The acquisition time of each spectrum was set to 30 s. Spectra were acquired from 3000 to 700 cm^−1^ with 2 cm^−1^ spectral resolution. Each spectrum is an average of at least 20 spectra obtained from various hot spots located at several (4–5) separately prepared samples.

### RAMAN measurements

Raman spectra of DNA-BLM complexes were recorded from samples containing the same molar ratio as described above; however, to obtain a sufficient signal-to-noise ratio, the final concentrations of DNA and BLM were higher than those in SERS samples. The concentration of DNA was 333.3 mg L^−1^. A droplet of the sample (1 µL) was transferred onto aluminium foil, and then the Raman spectra were immediately collected. The same Raman system as in the SERS experiment was used. Backscattered light was recorded using a 50 × objective with a laser power of 12.5 mW. All Raman spectra were acquired with a 20 s exposure time (2 accumulations) under excitation by a red (633 nm) laser. Each presented spectrum is an average of 5 spectra obtained from randomly chosen places on the sample.

### Spectral data processing

SERS and Raman spectra were baseline corrected (most of the spectra with 3rd order of polynomial, when necessary, the 3.2% of the spectra were corrected using the 5th order of polynomial), smoothed (Savitzky-Golay Filter, 5 smoothing points, 3rd polynomial order) and normalized (Standard Normal Variate) using MATLAB R2019b software (MathWorks, Inc., USA). When necessary, a cosmic ray removal procedure was applied. SERS spectra were mean and centred for partial least squares regression (PLSR) analysis in the fingerprint spectral range from 1600 to 700 cm^−1^, unique for biological molecules.

### AFM imaging

DNA samples for AFM imaging were prepared by DNA deposition on freshly cleaved mica (V1 grade, TED PELLA, INC, USA). Plasmid solution at a concentration of 0.625 mg L^−1^ in 10 mM HEPES buffer and 5 mM MgCl_2_ was used. A DNA solution was mixed with bleomycin to achieve BLM/DNA molar ratios from ca. 20–1000. Bleomycin was interacted with DNA for 4 min. The deposition time of the reaction solution was 3 min. Then, the sample was rinsed with 3 mL of distilled water and dried with a gentle flux of nitrogen^55^. To prepare AFM samples, DNA concentration and deposition time were carefully selected to avoid overlapping of individual DNA molecules. Molecules overlapping compromises the analysis of their length. Achieving the controlled surface coverage characterized by clear separation of individual molecules and the biggest possible surface coverage at the same time (to achieve sufficient statistics) is one of the biggest challenges in preparation of AFM samples. Moreover, it is extremely important to maintain surface coverage of AFM samples at the appropriate level, to enable the separation of studied molecules with applied AFM tip. Too short distance between molecules at the surface may result in a poor contrast.

AFM images were collected in air using a Multimode AFM Nanoscope IIIa system (Digital Instrument, Santa Barbara, CA, USA) in tapping mode. We used ETALON cantilevers (HA_NC, NT-MDT Spectrum Instruments). The force constant of the AFM cantilevers was from 3.5 to 12 N/m, and their resonance frequency was from 140 to 235 kHz. All the images were collected at a scan rate of 0.5 Hz, a scan frame of 512 × 512 pixels, and scan sizes of 3000 nm.

### Determination of the length of DNA fragments

The length of DNA molecules and their fragments (broken and circular—control) observed in AFM images were measured with FiberApp software^56^. Based on the length distributions, the total number of initial DNA molecules, N_0_, was determined for each sample as the sum of the number of broken plasmids, N_b_, and the number of unbroken plasmids, N_u_. N_b_ was determined by summing the lengths of the fragments measured and then dividing by the length corresponding to an unbroken plasmid,

$$N_{0} = N_{b} + N_{u} = \frac{{\sum\nolimits_{i = 1}^{n} {l_{i} } }}{L} + N_{u}$$

where *l*_*i*_ is the length of individual fragments in nanometres, *n* is the total number of fragments measured, and *L* is the length of an unbroken DNA molecule.

The mean DSB number was determined by dividing the total number of fragments (excluding unbroken plasmids) n by the total number of DNA molecules^50^.

$${\text{DSB}} = \frac{{\text{n}}}{{{\text{N}}_{{0}} }}$$

## Supplementary Information

Supplementary Information.

## Data Availability

The datasets used and/or analysed during the current study available from the corresponding author on reasonable request.
