# Supplementary material for: Plasmonic hot spots reveal local conformational transitions induced by DNA double-strand breaks
Source: Sci Rep. 2022 Jul 15;12:12158. doi: 10.1038/s41598-022-15313-4 (PMC9287445; doi:10.1038/s41598-022-15313-4)
Supplement: Supplementary file 1 — Supplementary Information. [file 41598_2022_15313_MOESM1_ESM.pdf]

SUPPLEMENTARY INFORMATION

Plasmonic Hot Spots Reveal Local Conformational Transitions Induced by DNA Double-Strand Breaks

Sara Seweryn, Katarzyna Skirlińska- Nosek, Natalia Wilkosz, Kamila Sofińska, David Perez-Guaita, Magdalena Oćwieja, Jakub Barbasz, Marek Szymoński, Ewelina Lipiec

Figure S1 A-I shows AFM images of pUC19 plasmid DNA interacting with Fe(III)-bleomycin complex solution and then adsorbed onto a mica surface. Several concentrations of BLM were applied.

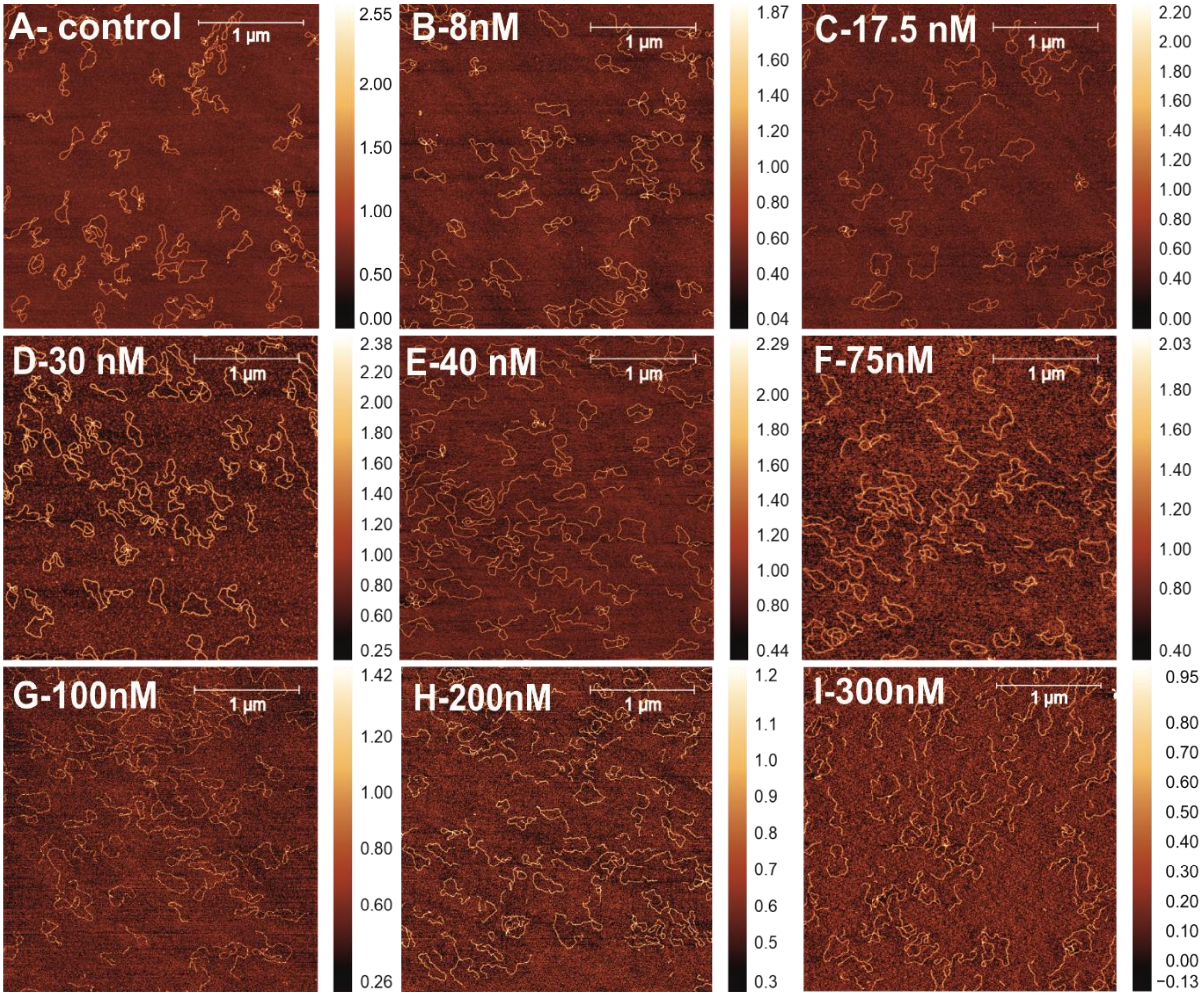

Figure S1. AFM imaging of pUC19 circular DNA plasmid damaged with bleomycin and fixed on mica: A) untreated DNA; B-I) DNA reacting for 4 mins with Fe(III)- bleomycin solution: linear fragments of various length are visible, the degree of fragmentation corresponds to increasing bleomycin concentration.

Table S1. Parameters for length distributions obtained based on AFM images of DNA treated with bleomycin.

**BLM concentration [nM]**	**Mean length of DNA fragments [nm]**	**Median**	**SD**
0	831	813	204
8	637	654	173
17.5	584	618	230
30	576	608	231
40	592	622	197
50	418	398	246
75	385	332	231
100	378	326	236
200	413	373	240
300	353	285	232

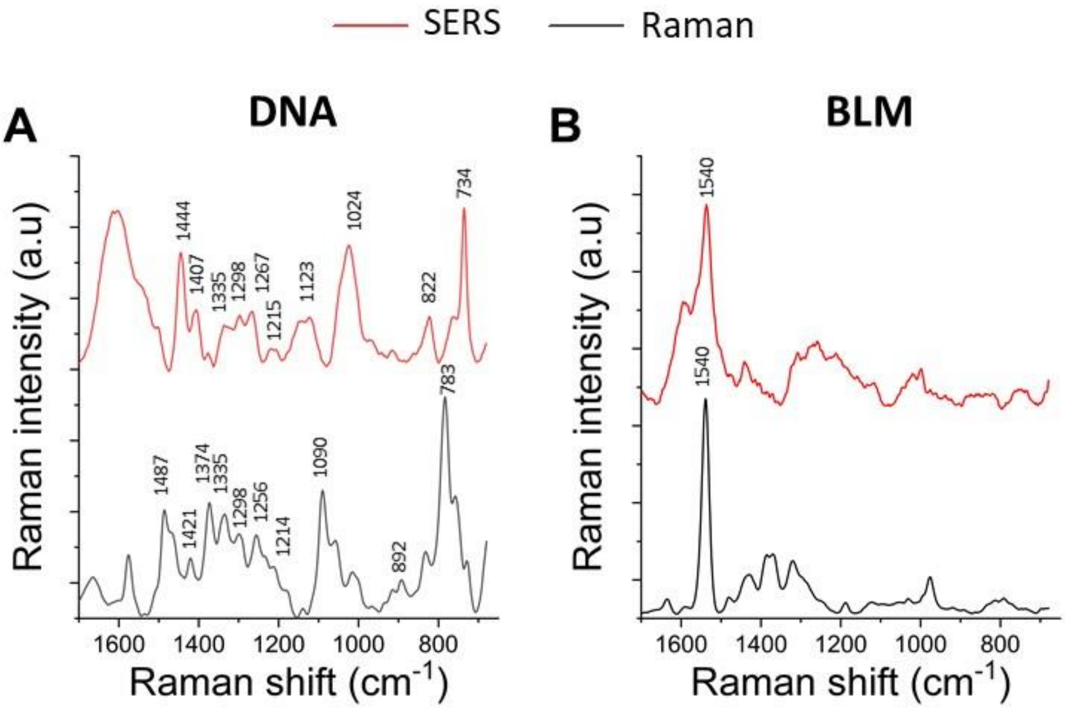

Figure S2. Raman **(black)** and SERS **(red)** spectra of (A) DNA**,** and (B) BLM.

Table S2. The assignment of Raman and SERS bands for DNA.

Band position [cm^{-1}]		Assignment
Raman	SERS	
1540	1540	Bithiazole moiety in BLM [1]
1487	1444	CH deformation vibration, ring breathing vibrations (A, G, C) [2]
1466	1407	C_5-CH_3 deformation vibration (T)[3]
1421	1376	Nucleobases (A)[2]
1374	1335	CH, CH_3 deformation vibration, C-N stretching (C,G)[2,4]
1335	1298	CH_3deformationvibration[5]
1298	1267	Ring breathingvibration (C)[3]
1256	1215	Asymmetric PO_2stretching[2,4,6]
1214	1123	Ring breathingvibration[5]
1090	1024	Symmetric PO_2stretching[2,6,7]
892	822	Asymmetric PO_2stretching[8,9]
783	734	C5′-O-P-O-C3′ phosphodiester bonds, nucleobases: A, T [9–11]

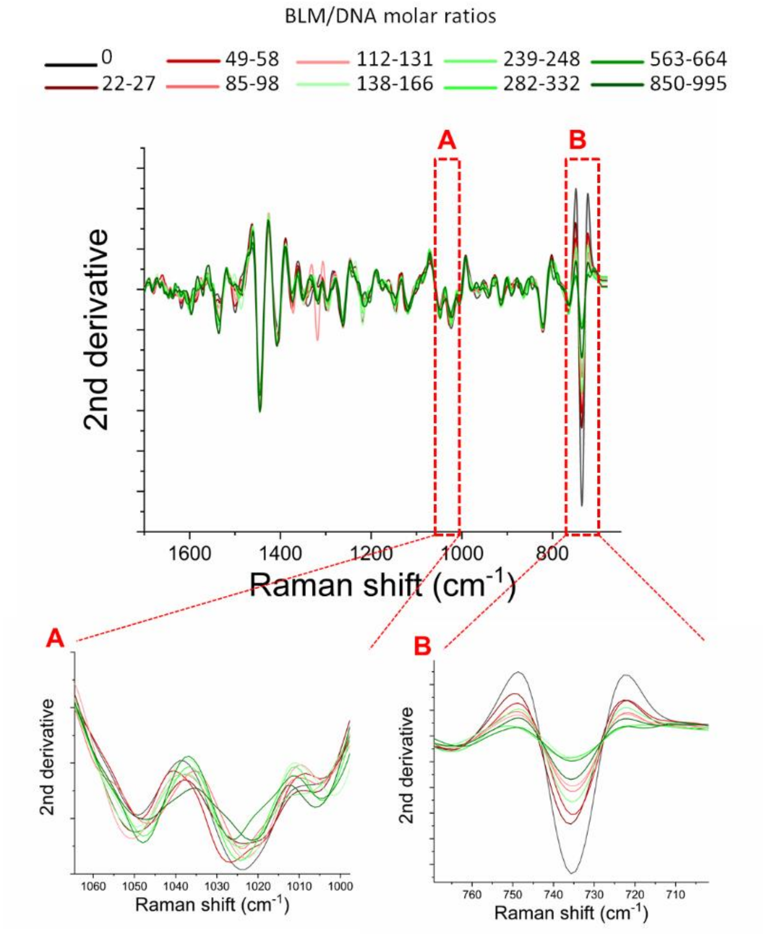

Figure S3. Second derivate of SERS spectra with zoomed areas of the spectral ranges of (A) symmetric stretching of phosphate at 1070 – 1000 cm^{-1}, (B) nucleobases (A,T) and C5’-O-P-O-C3′phosphodiester bonds band at 780 – 700 cm^{-1}.

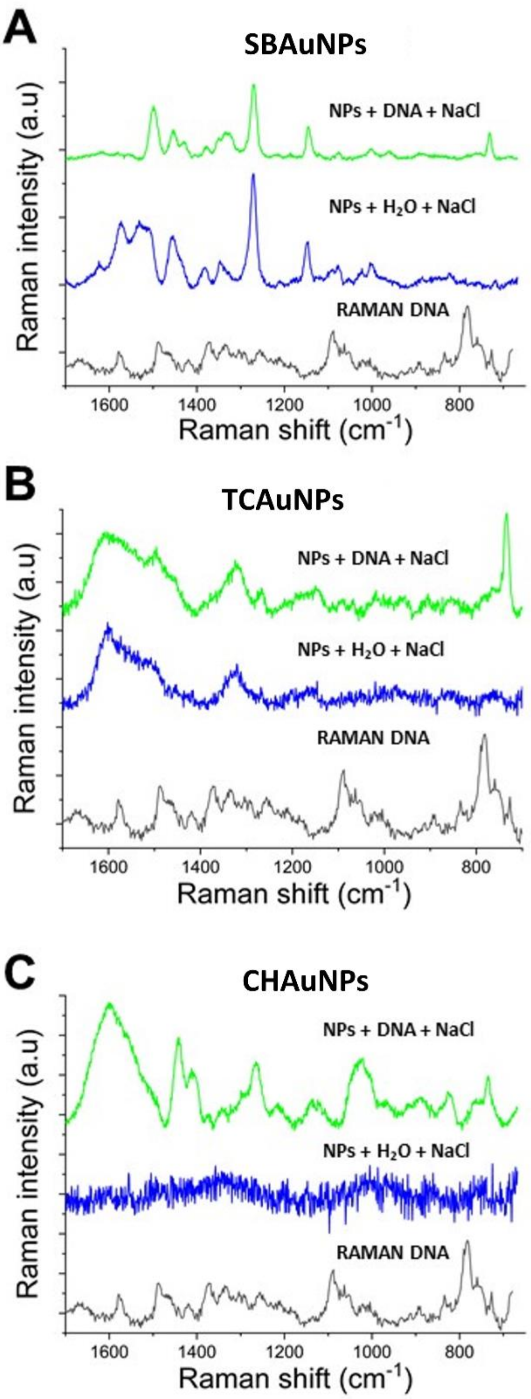

Figure S4. An optimization of SERS enhancer factor for efficient DNA measurement. SERS spectra of DNA were obtained using gold nanoparticles stabilized with (A) sodium borohydride, (B) trisodium citrate, (C) and cysteamine. For comparison, Raman spectra of

DNA, as well as SERS spectra of nanoparticles with stabilizers without DNA (acquired in analogical conditions) are presented.

Since gold nanoparticles are more stable and inert (less prone to oxidation) in physiological buffer than silver [12], gold nanoparticles were selected in these studies as the SERS substrate. The optimization of SERS technique for efficient DNA measurements considered examination of quality (SNR) of spectra achieved with the use of three different types of gold nanoparticles: stabilized with sodium borohydride (Fig. S4 A), trisodium citrate (Fig. S4 B) and cysteamine (Fig. S4 C). SERS spectra of nanoparticles with aggregating agent (0.05 M NaCl) and DNA were compared with the Raman spectra of concentrated DNA. For sodium borohydride nanoparticles, well-defined spectrum of stabilizer was obtained. Regarding that stabilizer itself is also clearly visible in the SERS DNA spectrum, these nanoparticles were not included in further measurements (Fig. S4 A). Comparing the Raman spectrum of concentrated DNA with SERS spectrum of DNA obtained with trisodium citrate nanoparticles, only one peak associated to phosphodiester bonds vibrations in DNA molecule at 734 cm^{-1} is well-resolved. As the SERS spectrum of DNA under selected conditions was not obtained, these nanoparticles were not considered in further studies as well (Fig. S4 B). For nanoparticles stabilized with cysteamine, well-resolved SERS spectrum of DNA was acquired with low influence of stabilizer itself (Fig. S4 C) and therefore these nanoparticles were selected for further optimization in terms of DNA concentration and concentration of the aggregating factor (Fig. S5).

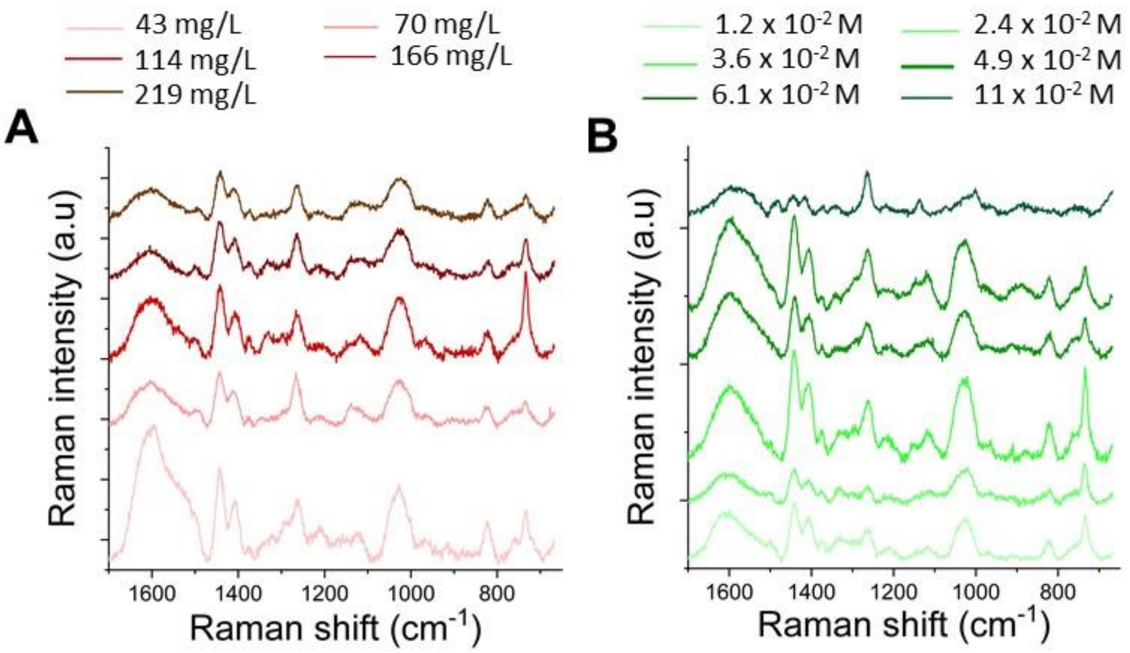

Figure S5. (A) SERS spectra of DNAat different concentrations of the analyte, (B) SERS spectra of DNA with different concentrations of aggregating agent.

Regarding the concentration of DNA in the sample, 114 mg L^{-1} was considered as the most appropriate due to the relatively low signal from stabilizer (the band from CH_2 torsion at 1266 cm^{-1}) in the acquired spectra (Fig. S5 A). Whereas for the ionic strength measurements, the concentration of 36 mM NaCl was selected as the SERS spectra of DNA collected under these conditions are characterized by the best signal-to-noise ratio and the highest intensity of the characteristic DNA Raman marker bands (Fig. S5 B). Before establishing final experimental parameters, number of additional optimization measurements were performed. We also examined the effect of sequence of experimental steps, and various incubation times (Fig. S6). Based on numerous systematic measurements, the most optimal experimental parameters were selected and then applied to the further studies of DNA damage.

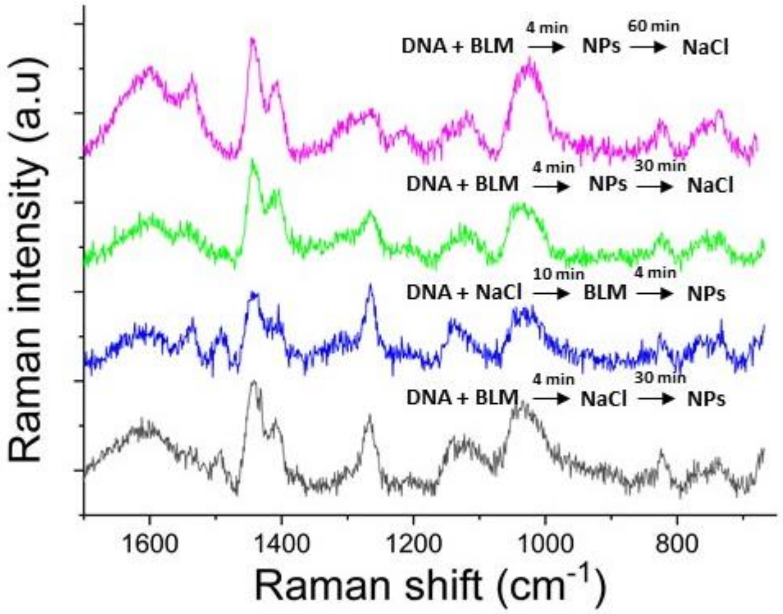

Figure S6. SERS spectra of DNA-BLM complex with different sequence of the particular components of the solution and time of the incubation.

Table S3. Assignment of Raman bands observed in SERS spectra and present in regression coefficient (β) plots calculated for PLSR models. The third column describes the VIP values [10,13].

Band position [cm^{-1}]	Assignment	VIP value
1645	deformation vibrations (thymine) [2]	0.91
1604	C-C stretches (imidazole ring) [14]	1.05
1577	C=N stretches (adenine) [2]	0.77
1537	DNA conformation marker (guanine related) [2]	1.22
1495	DNA conformation marker (guanine and adenine vibrations related) [2,15]	0.62
1463	($C5-CH_3$) deformation vibrations (thymine) [3]	0.89
1438	DNA Z-form conformation marker (adenine related) [2]	1.91
1416	symmetric CH, NH, CN stretches [2]	1.59
1391	CH_3 deformation vibrations (thymine) [2]	1.11

1361	ring breathing vibrations (thymine) [5]	0.87
1262	asymmetric PO_2 stretches [2]	1.24
1129	DNA Z-form conformation marker [2]	1.05
1098	symmetric PO_2 stretches [2]	1.06
1069	C-O stretches (deoxyribose) (enhanced in the case of the Z-form of DNA) [2]	1.37
970		0.88
951	C-C stretches (triplet determining the Z-form of DNA) [2]	0.84
925		0.75
809	thymine [13]	0.62
789	ring breathing vibrations (cytosine) [13]	0.77
728	adenine [13]	0.3

Synthesis of nanoparticles

Cysteamine-stabilized gold nanoparticles (CHSBAuNPs) were prepared according to the modified Niidome et al. [16] method. For this purpose, 1 mL of sodium borohydride solution (2.3 mM) (Sigma Aldrich) prepared in cold Milli-Q water was added dropwise to 50 mL of an aqueous solution of 1 mM tetrachloroauric(III) acid (Sigma Aldrich), which was mixed using a magnetic stirrer. Afterwards, 0.5 mL of a 1 mM solution of cysteamine hydrochloride (Sigma Aldrich) was added to the formed AuNPs.

Citrate-stabilized gold nanoparticles (TCAuNPs) were prepared according to modified Turkevich method [17] using trisodium citrate (Sigma Aldrich) as a reducing and stabilizing agent. Briefly, 200 ml of 1 mM tetrachloroauric(III) acid solution was heated up to 90°C. Afterwards, 10 ml of 75 mM trisodium citrate was added to the precursor solution. The reaction mixture was stirred vigorously (300 rpm) and heated over 20 minutes. After this period of time, the freshly prepared AuNP suspension was cooled in an ice bath.

Gold nanoparticles obtained with the use of sodium borohydride (Sigma Aldrich) (SBAuNPs) were prepared under ambient conditions via a step-by-step addition of 9 mL of sodium borohydride (SB) solution (10 mM) into 40 mL of gold(III) chloride acid trihydrate solution of

concentration of 1.5 mM. After the addition of sodium borohydride, the mixing of obtained suspension was continued for 30 minutes.

The ultrafiltration process, conducted according to the protocol described by Oćwieja et al. [18], was used for the purification of each type of obtained AuNP suspension.

Physicochemical characteristic of AuNPs dispersed in aqueous suspension.

The conductivity and pH of the stock AuNP suspension were measured using a multifunctional pH/conducto-meter (Elmetron). The concentration of AuNPs dispersed in the purified suspension was determined based on the density measurements described in detail elsewhere[19]. The optical properties of AuNPs dispersed in the suspensions of controlled pH, ionic strength and temperature were investigated based on the UV-vis spectra obtained using a UV-2600 spectrometer (Shimadzu). The morphology and size distribution of AuNPs were determined based on micrographs recorded using a JEOL JSM-7500F electron microscope. For this purpose, a drop of AuNP stock suspension was placed on a copper grid covered by a layer of carbon. After the evaporation of water, the sample was imaged using a transmission mode of the microscope. The size distribution was generated applying a MultiScan software (Computer scanning system). The stability and electrokinetic properties of cysteamine-modified AuNPs were evaluated under controlled conditions of pH, ionic strength and temperature using a Zetasizer Nano ZS instrument (Malvern). The hydrodynamic diameters (d_H) of AuNPs were calculated from the Stock-Einstein relationship knowing the diffusion coefficient (D). The Henry's model was applied for the determination of zeta potential (ζ) values of AuNPs.

The application of ultrafiltration method enabled to obtain AuNP suspension of a high purity. Despite of the AuNP preparation process was carried out under strong acidic condition, pH of purified stock suspension was equal to 5.8. The conductivity of suspension was attained a value of 3 $\mu S\ cm^{-1}$ which indicated on the lack of impurities coming from the unreacted reagents. It was established that the mass concentration of AuNPs in the stock suspension was 176 $mg\ L^{-1}$.

Extinction spectra recorded for the suspensions of concentrations of 150, 100 and 50 mg L^{-1} (Fig. S7A) showed that the AuNPs exhibit a plasmon absorption band at the wavelength of 520 nm. It is worth mentioning that these results are consistent with the findings obtained for other types of cysteamine-stabilized AuNPs described in the literature previously [18,20]. Additional experiment conducted for AuNP suspension under acidic (pH 3.5), neutral (7.4) and alkaline (9.0) conditions (Fig. S7 B) revealed that the pH changes influence on the position of plasmon absorption band. Significant bathochromic shift of plasmon adsorption band was observed for the AuNPs dispersed in alkaline suspension (pH 9.0).

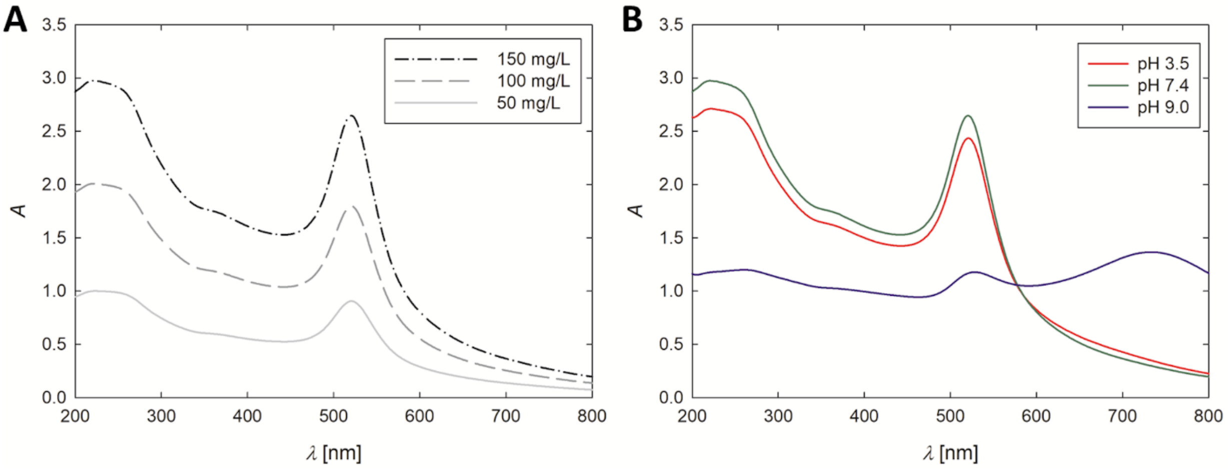

Figure S7. Extinction spectra of suspensions determined for (A) AuNP concentration of 150, 100 and 50 mg L^{-1}, at pH 5.8 and (B) for AuNP concentration of 150 mg L^{-1} at pH 3.5, 7.4 and 9.0.

Typical TEM micrograph of AuNPs was presented on Fig. S8 A. Analysing this image one can observe that cysteamine-stabilized AuNPs exhibit nearly spherical shape. Moreover, it was found that the AuNPs were fairly monodisperse (Fig. S8 B) and their average size was equal to 13±3 nm. This size value remains in agreement with the findings presented before [18] hence one can conclude that the preparation method of cysteamine-stabilized AuNPs is highly reproducible.

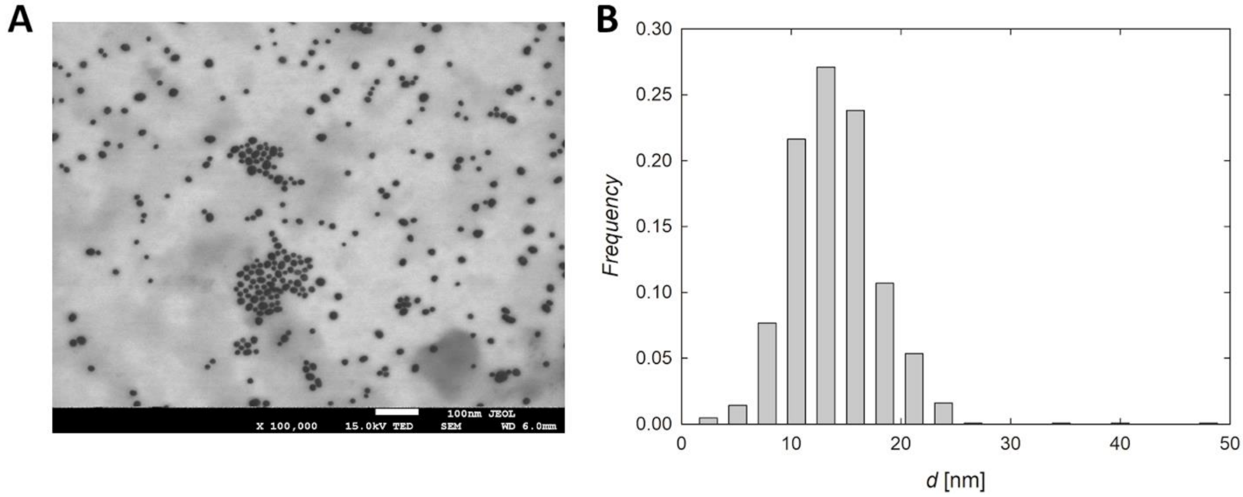

Figure S8. (A) TEM micrograph. and (B) size distribution of cysteamine-stabilized AuNPs.

The physicochemical characteristic of AuNPs dispersed in aqueous suspensions and conducted with the use of dynamic light scattering technique (DLS) revealed that the hydrodynamic diameter of nanoparticles dispersed in the stock solution was equal to 12±4 nm. Taking into account that the values of AuNP diameter determined applying TEM and DLS were comparable, it was concluded that the thickness of stabilizing layer formed from cysteamine molecules[18] is thin and negligible in comparison to the size of metallic core. The measurements of hydrodynamic diameter of AuNPs dispersed in the suspensions of controlled ionic strength and pH at the temperature of 25 ^{o}C showed that at pH 5.8 the aggregation process appeared for ionic strength higher than 10^{-2} M (Fig. S9 A). Independently on the ionic strength, the aggregation process of AuNPs leading to formation of aggregates of an average size of 348±24 nm was observed under alkaline conditions (for pH higher than 8) (Fig. S9 A) Thereby, the aggregation process of AuNPs detected by DLS technique explained the reason of bathochromic shift of plasmon absorption maximum under alkaline conditions (Fig. S7 B).

The electrokinetic properties of AuNPs were evaluated using electrophoretic scattering technique (ELS). It was established that the AuNPs were positively charged in board range of ionic strength and pH. The zeta potential of AuNPs dispersed in the stock solution at pH 5.8

was equal to 54±2 mV (Fig. S9 B). This value was comparable with the data described previously[18]. Similarly, the drop of zeta potential values with an increase of ionic strength and pH (Fig. S9 B) was also observed. It is worth mentioning that the aggregates of AuNPs formed at ionic strength range between 10^{-2} – $5x10^{-2}$ M were positively charged.

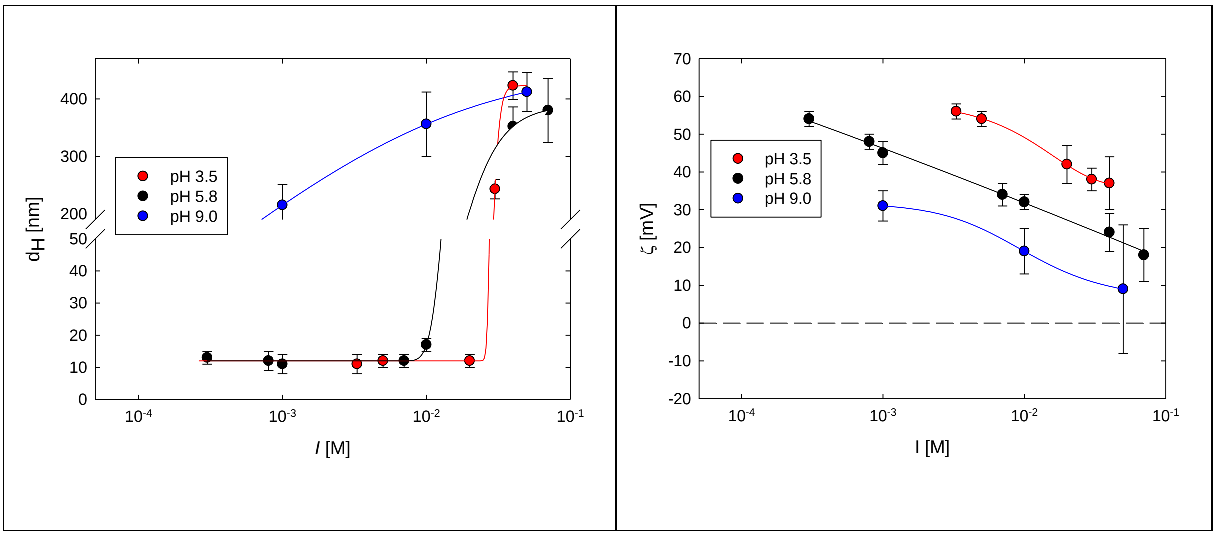

Figure S9. The dependence of (left) hydrodynamic diameter and (right) zeta potential of cysteamine-stabilized AguNPs on ionic strength determined at pH 3.5, 5.8 and 9.0. *T*=298 K and the suspension concentration of 50 mg L^{-1}. The solid lines present nonlinear fits of experimental data.

The synthesis of cysteamine-stabilized AuNPs was developed and described in numerous research articles. The production of cysteamine-stabilized AuNPs is conducted via a reduction of complexes formed by cysteamine and tetrachloroauric(III) acid molecules by strong reducing agents [16,20–29] or via a chemisorption of cysteamine molecules on the surfaces of previously formed AuNPs [18]. The cysteamine-stabilized AuNPs are well-known as effective substrates used in surface enhanced Raman spectroscopy (SERS) applied for detection of diverse organic and inorganic analyses. Previously, cysteamine-stabilized AuNPs and diverse cysteamine-stabilized nonspherical nanostructures were used for detection of perchloride anions [22], pentachloropentanol[30], sodium thiocyanate [31], benzoic acid [31], oxamyl and thiacloprid

pesticides [32], to name a few. However, to the best of our knowledge stable, spherical cysteamine-stabilized AuNPs dispersed in aqueous solution have not been applied for the detection of DNA double-strand breaks induced via bleomycin.

Utility of SERS/AFM methodology in tracking DNA conformational changes induced by various damaging factors

To demonstrate the utility of the approach presented in the manuscript (SERS combined with AFM), we performed experiments for genomic DNA isolated from Jurkat cells (30 kbp) treated with bleomycin (Fig S10) and pUC19 plasmid exposed to UVC radiation as a damaging factor (Fig S11). In both cases, damaging factors (BLM, UVC) induced DSBs (visible on AFM images), and conformational changes can be observed in the SERS spectra.

In the SERS spectra of pUC19 plasmid DNA exposured to UVC radiation, we have observed similar spectral changes as induced by BLM treatment including partial shift of the phosphate symmetric stretching from DNA backbone, which is a marker of DNA conformational change. A comparison of the spectra acquired from control and irradiated DNA shows the intensity decrease of the C5’-O-P-O-C3′ phosphodiester band at 850 – 680 cm^{-1} in the spectrum of DNA treated with UVC. This spectral change confirms the fragmentation of DNA strands with UVC.

Analogical spectral modifications can be observed in Fig S11, which demonstrates the results obtained for 30kbs genomic DNA isolated from Jurkat cells. This observation is consistent with the results described in the manuscript for pUC19 treated with BLM.

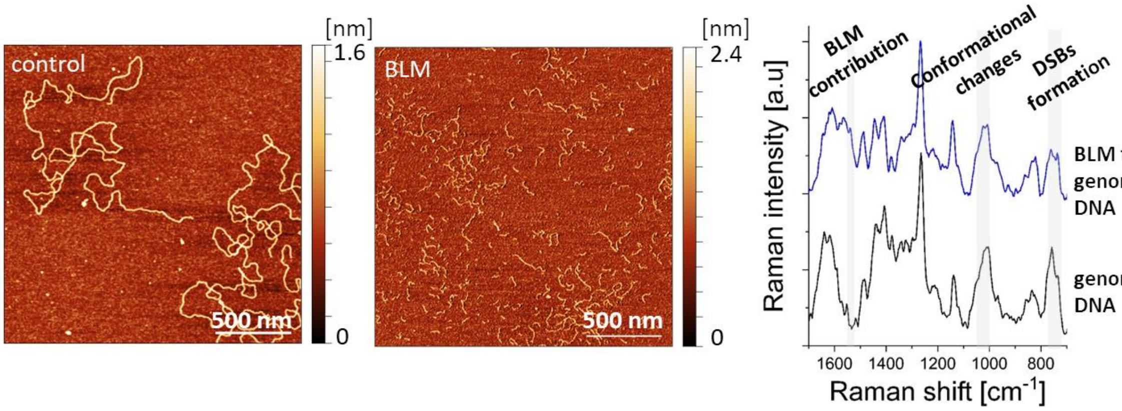

Figure S10. A comparison of AFM images and SERS spectra obtained from 30kbs genomic DNA isolated from Jurkat cells: control DNA and DNA treated with bleomycin.

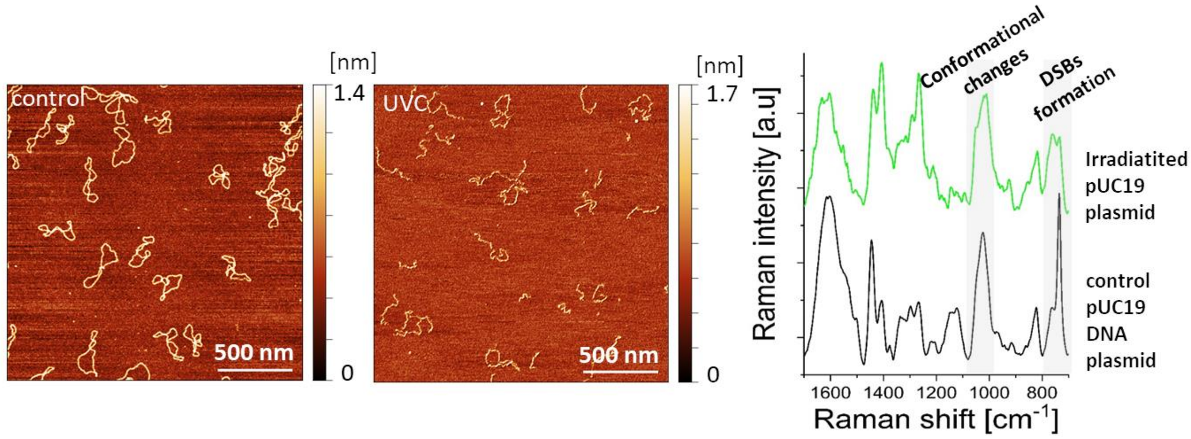

Figure S11. A comparison of AFM images and SERS spectra obtained from pUC19 plasmid DNA: control and treated with UVC.

SERS. For SERS investigation of BLM-induced molecular modifications in the genomic DNA isolated from Jurkat cells, 1.3 µL of Fe(III)-bleomycin (259.4 µM) was mixed with 1.2 µL of DNA solution (0.1 mg L^{-1}). After 4 minutes, a 2.5 µL AuNP suspension (209 mg L^{-1}) was added, and after 1 h of incubation, 0.7 µL NaCl (0.3 M) was added.

To investigate via SERS the molecular changes upon DNA after UVC irradiation, pUC19 DNA plasmid (500 mg L^{-1}) was diluted in MiliQ water at a ratio of 1.2 to 1.3, respectively. After 30

minutes of UVC radiation, 2.5 μL of the solution was mixed with 2.5 μL of AuNP suspension (209 mg L^{-1}) and after 1h, 0.7 μL NaCl (0.3 M) was added.

AFM imaging. DNA samples for AFM imaging were prepared by DNA deposition on freshly cleaved mica (V1 grade, TED PELLA, INC, USA). A solution of genomic DNA isolated from Jurkat cells at a concentration of 0.625 mg L^{-1} in 10 mM HEPES buffer and 5 mM $MgCl_2$ was used. A DNA solution was mixed with Fe(III)-bleomycin to achieve BLM/DNA molar ratio of 5000. BLM/DNA interaction time: 4 minutes. The deposition time of the solution was 3 minutes. Then, the sample was rinsed with 3 mL of MiliQ water and dried with a gentle flux of nitrogen.

Plasmid pUC19 solution at a concentration of 0.625 mg L^{-1} in 10 mM HEPES buffer and 5 mM $MgCl_2$ was used. After 30 minutes of UVC irradiation, the solution was deposited on freshly cleaved mica (3 minutes), and then rinsed with 3 mL of MiliQ water and dried with a gentle flux of nitrogen.

REFERENCES

1. Sam, J. W., Takahashi, S., Lippai, I., Peisach, J. & Rousseau, D. L. Sequence-specific changes in the metal site of ferric bleomycin induced by the binding of DNA. *J. Biol. Chem.* **273**, 16090–16097 (1998).
2. Banyay, M., Sarkar, M. & Gräslund, A. A library of IR bands of nucleic acids in solution. *Biophysical Chemistry* **104**, 477–488 (2003).
3. Ponkumar, S., Duraisamy, P. & Iyandurai, N. Structural Analysis of DNA Interactions with Magnesium Ion Studied by Raman Spectroscopy. *Am. J. Biochem. Biotechnol.* **7**, 135–140 (2011).
4. Dovbeshko, G. I., Gridina, N. Y., Kruglova, E. B. & Pashchuk, O. P. FTIR spectroscopy studies of nucleic acid damage. in *Talanta* **53**, 233–246 (Elsevier Science Publ Co Inc, 2000).
5. Escobar, R., Carmona, P., Rodríguez-Casado, A. & Molina, M. Raman spectroscopic determination of inosine nucleoside in nucleotides. in *Talanta* **48**, 773–780 (Elsevier, 1999).
6. Whelan, D. R. *et al.* Monitoring the reversible B to A-like transition of DNA in eukaryotic cells using Fourier transform infrared spectroscopy. *Nucleic Acids Res.* **39**, 5439–5448 (2011).
7. Wood, B. R. The importance of hydration and DNA conformation in interpreting infrared spectra of cells and tissues. *Chemical Society Reviews* **45**, 1980–1998 (2016).
8. Liquier, J. *et al.* Conformation of DNA in chromatin protein-DNA complexes studied by infrared spectroscopy. *Nucleic Acids Res.* **6**, 1479–1493 (1979).
9. Brown, E. B. & Peticolas, W. L. Conformational geometry and vibrational frequencies of nucleic acid chains. *Biopolymers* **14**, 1259–1271 (1975).
10. De Gelder, J., De Gussem, K., Vandenabeele, P. & Moens, L. Reference database of Raman spectra of biological molecules. *J. Raman Spectrosc.* **38**, 1133–1147 (2007).
11. Duguid, J. G., Bloomfield, V. A., Benevides, J. M. & Thomas, G. J. Raman spectroscopy of DNA-metal complexes. II. The thermal denaturation of DNA in the presence of Sr2+, Ba2+, Mg2+, Ca2+, Mn2+, Co2+, Ni2+, and Cd2+. *Biophys. J.* **69**, 2623–2641 (1995).
12. Solís, D. M., Taboada, J. M., Obelleiro, F., Liz-Marzán, L. M. & García De Abajo, F. J. Optimization of Nanoparticle-Based SERS Substrates through Large-Scale Realistic Simulations. *ACS Photonics* **4**, 329–337 (2017).

13. Tuma, R. Raman spectroscopy of proteins: From peptides to large assemblies. *J. Raman Spectrosc.* **36**, 307–319 (2005).
14. Stuart, B. H. *Infrared Spectroscopy: Fundamentals and Applications*. *Infrared Spectroscopy: Fundamentals and Applications* (wiley, 2005). doi:10.1002/0470011149
15. Benedetti, E., Bramanti, E., Papineschi, F., Rossi, I. & Benedetti, E. Determination of the relative amount of nucleic acids and proteins in leukemic and normal lymphocytes by means of fourier transform infrared microspectroscopy. *Appl. Spectrosc.* **51**, 792–797 (1997).
16. Niidome, T., Nakashima, K., Takahashi, H. & Niidome, Y. Preparation of primary amine-modified gold nanoparticles and their transfection ability into cultivated cells. *Chem. Commun.* **0**, 1978–1979 (2004).
17. Turkevich, J., Stevenson, P. C. & Hillier, J. A study of the nucleation and growth processes in the synthesis of colloidal gold. *Discuss. Faraday Soc.* **11**, 55–75 (1951).
18. Oćwieja, M., Maciejewska-Prończuk, J., Adamczyk, Z. & Roman, M. Formation of positively charged gold nanoparticle monolayers on silica sensors. *J. Colloid Interface Sci.* **501**, 192–201 (2017).
19. Oćwieja, M. & Adamczyk, Z. Monolayers of silver nanoparticles obtained by chemical reduction methods. *http://dx.doi.org/10.1680/si.13.00042* **2**, 160–172 (2015).
20. Luo, Y. *et al.* A novel colorimetric aptasensor using cysteamine-stabilized gold nanoparticles as probe for rapid and specific detection of tetracycline in raw milk. *Food Control* **54**, 7–15 (2015).
21. Ramírez-Segovia, A. S. *et al.* Glassy carbon electrodes sequentially modified by cysteamine-capped gold nanoparticles and poly(amidoamine) dendrimers generation 4.5 for detecting uric acid in human serum without ascorbic acid interference. *Anal. Chim. Acta* **812**, 18–25 (2014).
22. Ruan, C., Wang, W. & Gu, B. Surface-enhanced Raman scattering for perchlorate detection using cystamine-modified gold nanoparticles. *Anal. Chim. Acta* **567**, 114–120 (2006).
23. Lee, S., Bae, K., Kim, S., Lee, K. & Park, T. Amine-functionalized gold nanoparticles as non-cytotoxic and efficient intracellular siRNA delivery carriers. *Int. J. Pharm.* **364**, 94–101 (2008).
24. Sharma, A. *et al.* Antibody immobilized cysteamine functionalized-gold nanoparticles for aflatoxin detection. *TSF* **519**, 1213–1218 (2010).
25. Yun Jv, Baoxin Li & Rui Cao. Positively-charged gold nanoparticles as peroxidiase mimic and their application in hydrogen peroxide and glucose detection. *Chem. Commun.* **46**, 8017–8019 (2010).
26. Rui Cao & Baoxin Li. A simple and sensitive method for visual detection of heparin using positively-charged gold nanoparticles as colorimetric probes. *Chem. Commun.* **47**, 2865–2867 (2011).
27. Min Zhang, Yu-Qiang Liu & Bang-Ce Ye. Colorimetric assay for sulfate using positively-charged gold nanoparticles and its application for real-time monitoring of redox process. *Analyst* **136**, 4558–4562 (2011).
28. Liang, X. *et al.* Colorimetric detection of melamine in complex matrices based on cysteamine-modified gold nanoparticles. *Analyst* **136**, 179–183 (2010).
29. Ma, Y. *et al.* Colorimetric sensing strategy for mercury(II) and melamine utilizing cysteamine-modified gold nanoparticles. *Analyst* **138**, 5338–5343 (2013).
30. Jiang, X., Yang, M., Meng, Y., Jiang, W. & Zhan, J. Cysteamine-Modified Silver Nanoparticle Aggregates for Quantitative SERS Sensing of Pentachlorophenol with a Portable Raman Spectrometer. *ACS Appl. Mater. Interfaces* **5**, 6902–6908 (2013).
31. Hussain, A., Pu, H. & Sun, DW. SERS detection of sodium thiocyanate and benzoic acid preservatives in liquid milk using cysteamine functionalized core-shelled nanoparticles. *Spectrochim. Acta. A. Mol. Biomol. Spectrosc.* **229**, (2020).
32. Hussain, A., Pu, H. & Sun, D. W. Cysteamine modified core-shell nanoparticles for rapid assessment of oxamyl and thiacloprid pesticides in milk using SERS. *J. Food Meas. Charact.* **14**, 2021–2029 (2020).
